# Chatbots in Radiology: Current Applications, Limitations and Future Directions of ChatGPT in Medical Imaging

**DOI:** 10.3390/diagnostics15131635

**Published:** 2025-06-26

**Authors:** Ludovica R. M. Lanzafame, Claudia Gulli, Silvio Mazziotti, Giorgio Ascenti, Michele Gaeta, Thomas J. Vogl, Ibrahim Yel, Vitali Koch, Leon D. Grünewald, Giuseppe Muscogiuri, Christian Booz, Tommaso D’Angelo

**Affiliations:** 1 Diagnostic and Interventional Radiology Unit, BIOMORF Department, University Hospital “Policlinico G. Martino”, Via Consolare Valeria 1, 98100 Messina, Italy; 2 Department of Diagnostic and Interventional Radiology, University Hospital Frankfurt, Theodor-Stern-Kai 7, 60590 Frankfurt am Main, Germany; 3 Division of Experimental Imaging, Department of Diagnostic and Interventional Radiology, University Hospital Frankfurt, Theodor-Stern-Kai 7, 60590 Frankfurt am Main, Germany; 4 Department of Radiology, ASST Papa Giovanni XXIII Hospital, 24127 Bergamo, Italy

**Keywords:** artificial intelligence, radiology, chatGPT, large language models, natural language processing

## Abstract

Artificial intelligence (AI) is reshaping radiological practice, with recent advancements in natural language processing (NLP), large language models (LLMs), and chatbot technologies opening new avenues for clinical integration. These AI-driven conversational agents have demonstrated potential in streamlining patient triage, optimizing imaging protocol selection, supporting image interpretation, automating radiology report generation, and improving communication among radiologists, referring physicians, and patients. Emerging evidence also highlights their role in decision-making, clinical data extraction, and structured reporting. While the clinical adoption of chatbots remains limited by concerns related to data privacy, model robustness, and ethical oversight, ongoing developments and regulatory efforts are paving the way for responsible implementation. This review provides a critical overview of the current and emerging applications of chatbots in radiology, evaluating their capabilities, limitations, and future directions for clinical and research integration.

## 1. Introduction

The integration of artificial intelligence (AI) into radiology has markedly transformed various aspects of clinical workflow, from image acquisition to interpretation [1,2,3,4,5]. While much of the existing research has focused on imaging-based applications, recent advancements in natural language processing (NLP) and large language models (LLMs) have introduced new frontiers. Of particular interest is the emergence of chatbots, intelligent conversational agents capable of generating human-like text, which are increasingly being explored for their utility in radiology [6,7]. Although several reviews have previously addressed this topic, the rapid pace of research and continuous development of new models highlight the need for ongoing updates. This literature review aims to provide an in-depth and current overview of the existing and emerging applications of chatbot technologies, offering a comprehensive understanding of the technological landscape and evolving trends within the domain of radiology.

## 2. Literature Search Strategy

A narrative review was conducted to explore the use of chatbots and large language models in the field of radiology. Literature searches were performed using four major databases: PubMed, Google Scholar, Scopus, and Web of Science. The search covered publications from 2017 to 2025, using the following keywords: “*chatbot*” *OR* “*large language model*” *OR* “*GPT*” *AND* “*radiology*” *OR* “*imaging*”.

Eligible studies included peer-reviewed articles describing clinical or research applications of chatbots in radiology, encompassing both general discussions on large language models and studies focused on specific subspecialties within imaging. Non-English language articles and those lacking full-text access were excluded from the review.

All selected articles were critically assessed and synthesized to provide a comprehensive overview of current chatbot applications in radiology, their limitations, and potential future directions.

## 3. Natural Language Processing and Large Language Models

The NLP allows computers to understand and generate human language, forming the foundation for contemporary chatbot technologies [8]. The field has evolved from rule-based systems to neural-network-driven conversational agents.

The journey began in 1966 with ELIZA, a program based on scripted rules that used pattern matching, followed by incremental developments in conversational models such as PARRY, Jabberwacky, and ALICE, which, despite advances, lacked genuine contextual comprehension [9,10].

The emergence of voice-activated virtual assistants such as Apple’s Siri, Amazon Alexa, Microsoft Cortana, and Google Assistant represented a shift towards AI-powered personal assistants capable of addressing more complex queries [11,12]. A major leap occurred in 2017 with the introduction of the transformer architecture in the seminal paper “Attention Is All You Need” [13], leading to the creation of the generative pretrained transformer (GPT) models, which revolutionized the NLP landscape [14].

The public release of ChatGPT (OpenAI) in 2022, based on GPT-3.5, demonstrated the broad applicability of LLMs, from summarizing documents to generating executable code [15]. In 2023, the introduction of GPT-4 further improved performance, with enhancements in reasoning, factual accuracy, and multimodal capabilities (image and text processing), spurring competition and leading to the release of comparable models such as Claude 3 Opus (Anthropic), Gemini 1.5 Pro (Google), and GPT-4o (OpenAI) [16,17].

While these general-purpose models have shown impressive capabilities, there is growing interest in domain-specific LLMs tailored for healthcare. Several models, including BioGPT, PubMedBERT, GatorTron, Med-PaLM, and LLaVA-Med, have been developed to serve clinical and research needs more effectively [18]. Table 1 presents a terminology box summarizing the main LLM models referenced throughout this review.

## 4. Current Applications in Radiology

**Figure 1:**
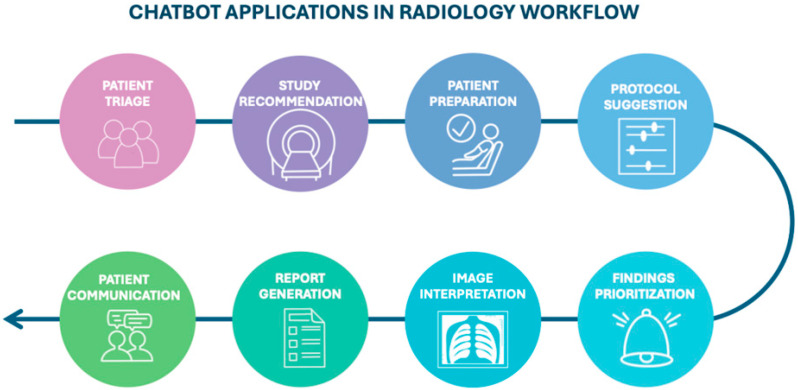
Diagram illustrating the potential stages of the radiology workflow where chatbots could be integrated, from patient triage to communication.

The increasing availability of chatbots and LLMs is accelerating their adoption in clinical radiology (Figure 1), where they can support a range of tasks.

### 4.1. Before Imaging: From Study Recommendation to Protocoling

The early phases of radiologic assessment, including study justification and protocol selection, are often time-intensive and vulnerable to variability. Radiologists frequently encounter incomplete or inappropriate referrals, leading to delays and inefficiencies. In this context, LLMs offer a promising tool for supporting imaging decision-making for clinicians [19].

Rau et al. demonstrated that chatbots can accurately recommend imaging modalities, performing comparably to radiologists while reducing both the time (2–8 min vs. 22–73 min) and cost of decision-making (both *p* ≤ 0.003) [20]. This functionality is particularly valuable in high-throughput settings such as emergency departments, where timely imaging decisions are critical [21].

Chatbots also serve as a patient education resource, offering tailored explanations of imaging indications, risks, and benefits, especially in complex scenarios such as imaging during pregnancy, contrast administration in renal impairment, and interventional procedures. While LLMs effectively convey accurate information, their simplification capabilities are currently limited and may require patients to possess a baseline level of health education [22,23,24,25].

Another emerging application is protocol selection optimization. Gertz et al. reported that GPT-4 achieved 84% agreement (95% CI: 75.3–90.6) with expert radiologists in choosing the correct imaging modality, region, contrast use and phase [26]. Moreover, AI can enhance the completeness of clinical data in requisition forms and assist in automated protocol recommendation, thereby improving the quality of radiologic interpretation [27].

### 4.2. Interpretation, Data Extraction and Diagnostic Capacity

The application of AI-powered chatbots in radiology has proven beneficial across several domains, including identifying radiological imaging findings, enhancing population screening programs, and reducing diagnostic errors [28,29]. ChatGPT has emerged as a potential tool for clinical decision support, particularly within neuroradiology and oncological imaging.

Horiuchi et al. investigated the use of ChatGPT-4.0 in neuroradiology imaging across various diseases. Their results suggested that integrating ChatGPT-4.0 into clinical workflows could enhance diagnostic accuracy, especially in metabolic, cerebrovascular, and neurodegenerative conditions. These improved outcomes were attributed to the presence of distinctive keywords in patient histories and imaging patterns. In contrast, diagnostic performance was lower in central nervous system tumors, likely due to their histopathological diversity and the rarity of some subtypes [30]. Notably, the study’s limitations included ChatGPT’s inability to directly process medical images and the absence of a direct radiologist comparison.

To address these limitations, Mitsuyama et al. evaluated GPT-4 in real-world clinical scenarios, focusing on brain tumor diagnosis. Their findings indicated that GPT-4 performed comparably to experienced neuroradiologists in providing differential diagnoses based on preoperative MRI reports, suggesting its potential as a supportive diagnostic tool for clinicians [31].

With the advent of GPT-4V, a vision-enabled model capable of both textual and visual analysis, future applications may extend into image-based diagnostics. A recent exploratory study by Kelly et al. examined the zero-shot performance of GPT-4V in detecting multiple sclerosis progression, comparing it to state-of-the-art vision models such as U-Net and Vision Transformer (ViT). Zero-shot learning (ZSL) is a machine learning paradigm that empowers models to identify novel objects, concepts, or diseases without having been explicitly trained on examples of those specific categories [32]. The study focused on identifying new white matter hyperintensities in fluid-attenuated inversion recovery (FLAIR) brain images. However, GPT-4V underperformed compared to the dedicated vision models, due to its inconsistencies and lack of interpretability, including misclassified cases and overly cautious non-answers [33].

Despite its multimodal potential, studies consistently indicate that GPT-4V’s diagnostic sensitivity and specificity remain inferior to domain-specific models [34,35].

Most recently, Koyun et al. evaluated the diagnostic performance of ChatGPT-4o for the detection and classification of intracranial hemorrhages on non-contrast CT. Their study highlights the potential of a general-purpose LLM in a specific medical imaging field, and its ability to classify hemorrhage type, stage, anatomical location, and associated findings. However, the model struggled with detecting certain hemorrhage types, such as subarachnoid hemorrhage [36].

Progress has also been made in exploring LLMs for oncological applications. A study published in Patient Education and Counseling evaluated ChatGPT’s alignment with the United States Preventive Services Task Force (USPSTF) guidelines for breast and prostate cancer screening. While responses generally adhered to evidence-based recommendations, discrepancies emerged in scenarios involving older patients, emphasizing the continued necessity of human oversight and clinical judgement [37].

Beyond screening recommendations, the use of large language models, particularly focusing on transformer-based models as Bidirectional Encoder Representations from Transformers (BERT) have demonstrated robust performance in clinical information extraction [38]. Hu et al. developed a deep learning pipeline for automated extraction of lung cancer staging parameters from CT reports based on TNM classification. Their model showed high accuracy in identifying tumor size, lymph node involvement, and metastatic status, streamlining the staging process and reducing manual workload [39].

Similarly, Zhou et al. [40] developed CancerBERT, a fine-tuned BERT model enriched with oncology-specific vocabulary. It reliably extracted eight key attributes, including tumor grade, histological type, and stage, thereby facilitating structured staging for breast cancer patients [40].

Building on these developments, Fink et al. [41] evaluated GPT-4’s accuracy in extracting oncological information from unstructured CT reports in lung cancer. When guided by predefined prompts, GPT-4 demonstrated improved performance over previous versions of ChatGPT, particularly in identifying metastatic spread, and tracking disease progression [41].

However, persistent challenges with hallucinations and referencing accuracy have limited LLM adoption for high-stakes clinical tasks. Recent developments in retrieval-augmented generation (RAG) models aim to address these concerns. By linking model output to externally validated knowledge sources, RAG-enhanced LLMs offer improved factual accuracy and contextual grounding [42]. Tozuka et al. [42] evaluated NotebookLM, a RAG-based LLM, for lung cancer staging. Using national staging guidelines as the retrieval base, NotebookLM staged 100 hypothetical cases from CT findings, outperforming GPT-4o and underscoring its potential for structured image-based decision-making [42].

### 4.3. Chatbots and Report Generation

A key application of LLMs in radiology is their potential to enhance clinical workflows by supporting radiology report generation. These reports typically comprise three major components:

- The “indication”, which outlines the clinical context and rationale for the examination.
- The “findings section”, which documents the radiologist’s observations from imaging data.
- The “impression”, which synthetizes key findings to inform potential diagnoses or management recommendations.

Radiologists must integrate imaging findings with clinical information to generate accurate and actionable impressions. Recent studies have demonstrated that LLMs, such as ChatGPT, can generate diagnostic impressions when provided with imaging findings, either in the form of structured data or narrative text.

LLMs can potentially reduce the cognitive workload associated with drafting diagnostic impressions, particularly in complex cases involving ambiguous or overlapping imaging findings. Impression generation is a crucial step in radiologic interpretation, guiding downstream clinical decision-making. Conventionally, this task is completed manually by the radiologist, which is time-intensive and prone to variability.

Although fine-tuned models can automate impression drafting, they typically require extensive annotated datasets—resources that are often scarce in subspecialties such as interventional, oncological, or neuroradiology. To address this, Sun et al. [43] systematically evaluated GPT-4’s performance in zero-shot impression generation. The model’s outputs were compared with radiologist-authored impressions using four key criteria: coherence, comprehensiveness, factual accuracy, and potential for harm. Results indicated that while GPT-4 exhibits promising capabilities, it does not yet achieve parity with human-generated impressions, underscoring the need for further model refinement and clinical validation before routine use [43].

Another active area of investigation concerns the capacity of LLMs to deliver tailored clinical recommendations. A study published in European Radiology compared ChatGPT’s performance in imaging appropriateness with that of a validated clinical decision support system (CDSS), the ESR iGuide. While ChatGPT demonstrated reasonable accuracy in recommending imaging tests, it lacked integration with electronic health records and could not provide fully personalized advice. Its reliance on statistical pattern recognition, rather than individualized reasoning, limits its current clinical applicability in this domain [44].

Similarly, Truhn et al. [45] explored GPT-4’s performance in recommending orthopedic treatments based on MRI findings. While the model generated largely appropriate recommendations, it occasionally failed to grasp the urgency or clinical context, instead producing generalized suggestions of limited utility in nuanced cases [45]. In pediatric radiology, LLMs like ChatGPT have been considered as adjunct tools to support decision-making, particularly where imaging guidelines are complex or age-specific. In oncology, a recent study in npj Breast Cancer evaluated ChatGPT-3.5 as a virtual participant in breast tumor board discussions. While the model was effective at summarizing case information and explaining the rationale, it was less proficient in issuing clinical recommendations, reinforcing the current role of LLMs as supportive, rather than autonomous tools [46].

#### Structured Reporting

Structured reporting represents another promising application for LLMs in radiology. It enhances clarity, facilitates data retrieval, and promotes standardization of terminology across institutions. However, transitioning large volumes of free-text reports into structured templates remains a logistical challenge.

Adams et al. [47] explored the feasibility of using GPT-4 to automate this process across multiple imaging modalities (CT, MRI, chest radiography) and languages. Their findings suggest that GPT-4 can reliably extract relevant content and reformat it according to predefined templates, although challenges related to language variability and contextual nuance require further investigation [47].

In a complementary study, Grewal et al. [48] evaluated GPT-4’s ability to generate radiology report templates through NLP. The model successfully extracted demographic data, clinical history, anatomical details, and imaging findings from narrative reports and translated them into structured formats. This capability has implications not only for standardizing reporting practices but also for supporting educational initiatives and decision-making, provided that ethical and legal considerations are carefully addressed [48].

### 4.4. Role of Chatbots in Patient Communication

Radiology reports are typically written using technical and specialized terminology intended for healthcare professionals, which can create a significant communication barrier for patients. This complexity often leads to confusion, anxiety, and misinterpretation, particularly when patients access their imaging reports through electronic health record portals prior to consultation with their referring physician.

LLMs have demonstrated the potential to address this challenge by translating radiological findings into layperson-friendly summaries. These AI-generated interpretations can enhance patient understanding by conveying key information with high levels of factual accuracy and completeness [49,50]. By improving health literacy and reducing ambiguity, LLMs may facilitate more effective communication between patients and healthcare providers and foster greater patient engagement in clinical decision-making.

Nevertheless, significant concerns remain regarding the reliability of AI-generated explanations. Inaccurate or misleading simplifications can have serious consequences, potentially leading to inappropriate patient actions, emotional distress, or the erosion of trust in the healthcare system. Therefore, ensuring that AI-generated content maintains clinical accuracy and conveys nuance appropriately is essential for safe integration into patient-facing communication tools [51,52,53].

A study by Jeblick et al. underscores these risks, revealing that approximately one-third of simplified radiology report summaries contained factual inaccuracies or misrepresentations [54].

These findings highlight the critical need for careful validation, human oversight, and transparent risk communication when deploying LLMs for patient education purposes.

Table 2 provides a comprehensive overview of the role of advanced LLMs across the various stages of the radiology workflow, detailing their potential applications while also critically addressing the limitations associated with each specific use case.

## 5. Performance of Contemporary Chatbots

Developing a dedicated specialist AI model for each clinical task is often impractical due to the wide-ranging complexity of healthcare environments and the resource-intensive nature of model development. Although relatively few comparative studies of LLMs have been conducted to date, early evidence reveals important performance distinctions across platforms.

In a study evaluating responses to non-expert-level questions on lung cancer, including topics such as prevention, screening, and basic radiology terminology, ChatGPT-3.5 demonstrated superior accuracy compared to Google’s Bard. However, neither model achieved full consistency or reliability across all tasks, underscoring ongoing limitations in generalist chatbot performance [55]. Similarly, in tasks involving radiology report simplification, both ChatGPT-3.5 and ChatGPT-4 outperformed Bard and Bing Chat, particularly in producing summaries that were more coherent, accurate, and accessible to lay readers [56].

In cardiovascular imaging, Silbergleit et al. [57] conducted a comparative analysis of four general-purpose LLMs—ChatGPT-3.5, ChatGPT-4, Google Gemini, and Gemini Advanced—in the automated generation of coronary artery disease reporting and data system (CAD-RADS) scores and recommendations based on coronary CT angiography (CCTA) reports. CAD-RADS scoring is essential for standardized risk assessment and clinical decision-making in cardiac imaging. Among the evaluated models, ChatGPT-4 demonstrated the highest accuracy and concordance with radiologist-generated scores, followed by Gemini Advanced. Although ChatGPT-3.5 showed faster response times, its accuracy was inferior to newer models. These findings reinforce the clinical potential of LLMs while emphasizing the continued need for large, high-quality annotated datasets and significant computational resources for optimal performance [57].

In neuroimaging, Koyun et al. [28] evaluated Claude 3.5 Sonnet in the detection of acute ischemic stroke using diffusion-weighted imaging (DWI). Claude 3.5 outperformed ChatGPT-4 in both lesion localization and agreement with expert radiologists, highlighting the rapid advancements and nuanced capabilities of emerging LLMs in image interpretation tasks [28].

In oncology, a recent study explored the use of GPT-4 to automate TNM staging for lung cancer based on unstructured CT reports. Using advanced prompt engineering to guide GPT-4 according to formal staging criteria, the model significantly outperformed GPT-3.5 Turbo, demonstrating higher accuracy and consistency. These findings illustrate the expanding role of LLMs in streamlining complex oncological workflows and supporting data abstraction for clinical decision-making [58].

It is important to acknowledge the pace at which LLMs are evolving, making it difficult to predict which platform may emerge as the most suitable or dominant in a clinical setting [39]. Given the heterogeneity of radiological tasks and their varying degrees of complexity, radiologists must critically assess whether to deploy general-purpose LLMs, domain-adapted generalist models, or bespoke specialist systems. Such strategic selection is essential to ensure optimal model performance, safety, and clinical relevance.

## 6. Limitations

LLMs have demonstrated strong performance when guided by structured prompts, serving as virtual assistants for radiologists and other healthcare professionals in a variety of tasks. However, their integration into clinical decision-making continues to be limited by several critical shortcomings.

**Figure 2:**
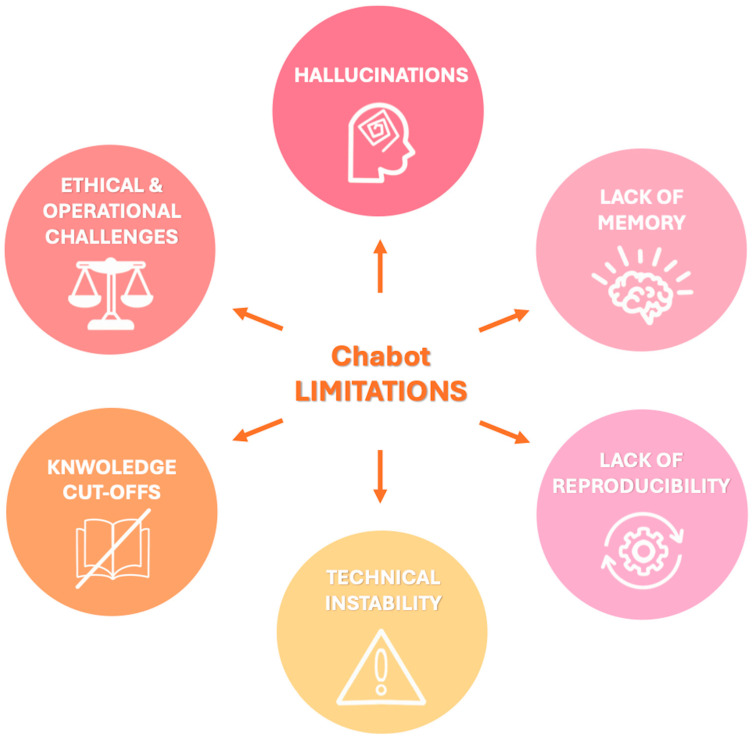
Visual summary of key limitations associated with chatbot integration in radiology, that should be carefully considered to ensure safe and effective implementation in clinical workflows.

The principal limitations can be summarized as follows and are represented schematically in Figure 2:

- *Hallucinations*: LLMs may generate factually incorrect or fabricated content, a phenomenon known as “hallucination” since they generate responses based on patterns learned during training rather than verified data. For example, when asked about non-existent Lung-RADS categories, both ChatGPT and Bard gave confident but incorrect answers rather than indicating the absence of such categories [59]. The dissemination of inaccurate information by chatbots can have serious consequences for patient care, impacting not only clinical decision-making but also the psychological well-being of patients with increased emotional distress which has been linked to an elevated risk of suicidal behavior, especially among adolescents and young adults following a cancer diagnosis [55,59,60]. To mitigate hallucinations and improve factual consistency, recent developments have explored RAG models [48]. These systems combine LLMs with external information retrieval capabilities, enabling them to cite verifiable sources and improve reliability. For instance, a recent proof-of-concept study introduced the Gastrointestinal Imaging-Aware Chatbot (GIA-CB), a GPT-4–based model enhanced with domain-specific retrieval. When applied to abdominal imaging cases, GIA-CB outperformed standard GPT-4 in suggesting accurate differential diagnoses, demonstrating the value of domain-informed RAG systems [61].
- *Lack of memory*: Current LLMs do not have persistent memory of prior interactions. Each response is generated independently, without continuity, historical awareness, or contextual integration across sessions. Ongoing research is exploring LLMs with memory-enhanced architectures capable of retaining structured interaction histories and implementing a two-tier memory system—modeled after human short-term and long-term memory—to preserve context and ensure continuity throughout conversations [62]. Another promising approach involves developing longitudinal interaction-aware models that support session continuity or interface with electronic health records (EHRs) to maintain contextual coherence across multiple encounters [63].
- *Lack of reproducibility*: Responses to the same prompt may vary upon repetition due to the models’ stochastic and non-deterministic nature. While this enhances linguistic versatility, it limits credibility and undermines consistency in clinical applications [38]. To enhance the reproducibility of LLM outputs, several strategies have been employed to reduce response variability, including prompt engineering, modifying sampling techniques (e.g., nucleus sampling), and fixing the random seed; notably, setting the temperature parameter to 0.0 in conjunction with a fixed seed has been shown to produce more deterministic responses and minimize outlier benchmark scores, thereby improving the reliability and interpretability of evaluation results [64].
- *Technical instability*: ChatGPT and similar systems may experience latency or interruptions during periods of high usage, potentially delaying critical information retrieval [65].
- *Knowledge cutoffs*: LLMs are limited to the data available at the time of their last training update. They are not capable of incorporating newly published medical evidence, evolving guidelines, or real-time clinical data, resulting in potentially outdated or misleading recommendations [48]. One promising solution to this limitation is the integration of lifelong learning capabilities, which aim to enable LLM-based agents to continuously acquire, retain, and apply new knowledge over time, improving their adaptability to evolving medical contexts and enhancing the relevance and accuracy of their outputs in dynamic clinical environments [66].

In addition to these technical limitations, several ethical and operational challenges must be addressed:

- *Data privacy*: The use of AI tools in radiology often involves handling sensitive patient data. Reliance on cloud-based public platforms introduces risks of data breaches and unauthorized access. A potential solution is to use open-source LLMs within hospital networks. Since their source code is publicly available, they can be downloaded and implemented locally, helping to improve data security, ensure system availability, and promote transparency [67].
- *Transparency and traceability*: Most LLMs do not provide source references for their responses, complicating validation and undermining clinical trust. Transparency in AI systems requires clear communication about what the system can and cannot do, its purpose, the conditions under which it performs reliably, and its expected accuracy. This information is vital for healthcare providers, but also for patients, who should be informed when AI is involved in their care. Dealing with these ethical challenges in AI will require a multidisciplinary approach: combining technical safeguards, government regulation, oversight mechanisms, and collaborative ethical standards developed with input from clinicians, patients, AI developers, and ethicists [68,69].
- *Monopolization of knowledge*: The rapid development of LLMs has been dominated by a few large commercial entities (e.g., OpenAI/Microsoft, Google, Meta). To avoid the centralization of medical knowledge and preserve equitable access to AI technologies, support for non-commercial, open-source medical LLMs is crucial [14].
- *Regulatory oversight*: The absence of standardized policies regarding AI deployment, accountability, and error management poses risks to patient safety. Robust regulatory frameworks are necessary to guide safe and ethical AI integration into radiological workflows [48].

The integration of AI-driven chatbot technologies into radiology workflows presents a range of complex challenges, spanning technical infrastructure, interoperability, workforce training, change management, and financial investment. Technologically, many radiology departments rely on legacy Information Technology (IT) systems that are not readily compatible with modern AI tools, often requiring substantial upgrades or reconfiguration. Achieving seamless interoperability between chatbots and existing systems such as Picture Archiving and Communication Systems (PACS) and Radiology Information Systems (RIS) demands strict adherence to healthcare data standards, including DICOM and HL7, and often involves addressing vendor-specific integration issues. A comprehensive review by the Radiological Society of North America (RSNA) underscores the critical importance of standards-based interoperability to facilitate scalable AI adoption within radiology workflows [70].

From an organizational standpoint, the successful deployment of AI tools hinges on robust training initiatives. Radiologists and allied healthcare professionals must be equipped not only to operate these models but also to critically assess and interpret their outputs. This necessitates customized training programs and continuous professional development. Furthermore, effective-change management is essential to mitigate resistance and support staff as they adapt to AI-enhanced workflows [69].

Financially, the upfront investment required to implement chatbot systems, including infrastructure upgrades, software integration, and personnel training, must be carefully weighed against potential long-term gains. However, the realization of these benefits is highly context-dependent and should be evaluated through rigorous, institution-specific cost-benefit analyses [71,72].

It is worth highlighting that this review does not specifically investigate the underlying technical biases of LLMs, a topic that lies predominantly within the fields of computer science and engineering, although such limitations may nonetheless exert a significant influence on their performance and reliability in radiological applications.

## 7. Future Directions

As LLMs continue to evolve, their integration into radiology will increasingly span both clinical practice and research (Table 3).

The development of domain-specific medical LLMs, with enhanced contextual comprehension, domain-adapted vocabulary, and integration into electronic health record systems, could significantly enhance their clinical utility. Such integration may also address persistent concerns related to data privacy and regulatory compliance, provided that robust data governance and security protocols are implemented.

In subspecialties like interventional radiology, chatbots could be developed to assist with real-time procedural guidance and patient management. For example, a chatbot integrated with clinical decision support systems could provide instant recommendations during image-guided interventions—such as suggesting optimal access routes or alerting about potential complications based on patient data and imaging [73].

Embedding LLMs within PACS or RIS systems may enable automated alerting for critical findings or recommend next steps based on guidelines.

Beyond clinical utility, LLMs also hold transformative potential in the domain of medical research and education. These tools can democratize access to scientific literature, streamline scientific writing, and assist in complex computational analyses. By enabling researchers to efficiently navigate large datasets, extract relevant information, and generate preliminary drafts of scientific content, LLMs may reduce administrative burden and enhance research productivity [14]. However, the risk of misinformation propagation, the potential for overreliance on AI-generated content, and the necessity for human oversight in scientific authorship and interpretation remain significant. Furthermore, the importance of reproducibility, proper source attribution, and methodological transparency must not be overlooked [74].

LLMs such as ChatGPT have demonstrated near-passing performance on radiology board-style exams without image inputs, particularly excelling in lower-order reasoning and clinical management tasks. However, their performance declines on higher-order questions requiring image interpretation, complex reasoning, or conceptual application. As these models are increasingly integrated into radiology education to simulate board scenarios, generate differential diagnoses, or quizzes, it is essential to leverage their capabilities to support learning, while preserving the development of critical thinking, clinical judgment, and communication skills [75,76].

Although precise timelines remain difficult to predict, initial uses of LLMs in radiology, particularly in supportive or administrative contexts, may emerge gradually in the near future. Wider clinical adoption is likely to follow over time, as technical and regulatory challenges are gradually addressed, with the pace and cost-effectiveness of implementation varying depending on institutional resources, potentially leading to significant discrepancies between large and small centers.

## 8. Conclusions

Large language models such as ChatGPT demonstrate considerable promise in transforming radiological practice by enhancing workflow efficiency, supporting diagnostic reasoning, and facilitating research innovation. Nonetheless, their clinical deployment must be guided by ethical oversight, awareness of model limitations, and a clear framework for human supervision.

As patient-facing applications such as ChatGPT become more accessible, new challenges may emerge, including the risk of self-interpretation of radiological findings and potential miscommunication. These concerns underscore the importance of maintaining the interpretative role of radiologists and the necessity of professional oversight.

With ongoing model refinements and deeper integration into radiology-specific workflows, ChatGPT and similar tools may play a pivotal role in augmenting radiological services and improving patient outcomes [65]. Realizing this potential will require sustained interdisciplinary collaboration among AI developers, radiologists, and healthcare policymakers to ensure responsible implementation, regulatory alignment, and clinical relevance. At present, these tools should be regarded as supportive adjuncts, designed to assist rather than replace the clinical expertise, contextual reasoning, and ethical judgment of radiology professionals [44].

## Figures and Tables

**Table 1:** Summary of key foundation models and tools used in radiology and biomedical domains, highlighting their developers, core capabilities, and clinical relevance.

Model/Tool	Description
**GPT-4o**	Multimodal model capable of processing and generating text, images, and audio in real time. GPT-4o offers faster performance and reduced costs compared to its predecessors.
**GPT-4V**	Extension of GPT-4 with vision capabilities, enabling the model to analyze and interpret image inputs.
**ChatGPT-3.5**	Text-based model based on GPT-3.5 architecture, known for its proficiency in general reasoning tasks but with limitations in handling complex queries.
**Claude 3.5 Sonnet**	Mid-tier model in the Claude 3.5 family, optimized for reasoning and coding tasks, offering a balance between performance and efficiency.
**Claude 3 (Opus, Sonnet, Haiku)**	Family of large language models with varying capabilities, supporting extended context windows and multimodal inputs.
**Gemini 1.5 (Pro, Flash)**	Multimodal models with extended context lengths up to 1 million tokens. Pro offers comprehensive capabilities, while Flash is optimized for speed.
**Google Bard**	Conversational AI interface initially powered by PaLM 2 and later integrated with Gemini models, facilitating interactive dialogues and information retrieval.
**NotebookLM**	AI-powered research assistant designed to help users organize, summarize, and generate content from their documents, enhancing productivity and comprehension.
**Med-PaLM 2**	Medical-domain-tuned version of PaLM 2, achieving expert-level performance on medical question-answering benchmarks such as MedQA.
**BioGPT**	Domain-specific LLM trained on biomedical literature, effective in biomedical named-entity recognition, question answering, and text-generation tasks.
**PubMedBERT**	Transformer model pretrained from scratch on PubMed abstracts and full texts, tailored for biomedical natural language processing tasks.
**CancerBERT**	BERT-based model trained on oncology-specific literature and electronic health records, designed for cancer-related text mining and analysis.
**Clinical Camel**	Open biomedical language model trained on clinical dialogues and notes, aiming to democratize access to clinical AI tools.
**GatorTron**	LLM trained on extensive clinical records and notes, targeting medical summarization, concept extraction, and decision support applications.
**LLaVA-Med**	Vision–language model fine-tuned on medical images and captions, adapted from LLaVA for medical visual question answering and image-captioning tasks.

**Table 2:** Benefits and limitations of advanced LLMs across key radiology workflow stages.

Workflow Stage	Application Area	Potential Benefits	Limitations
**Before Imaging**	Study recommendation Patient education Protocol optimization	Enables rapid and cost-efficient decision-making Provides tailored patient explanations about indications, benefits, and risks Improves protocol selection and precision	Requires clinical oversight for accuracy and patient safety Assumes a baseline level of patient literacy
**Interpretation, Data Extraction, and Diagnostic Capacity**	Data interpretation Clinical information extraction Enhanced diagnostic accuracy	Enhances diagnostic accuracy by extracting relevant clinical information from images Enables simultaneous visual and textual interpretation (multimodal capabilities) Enables comparable performance to expert radiologists (e.g., GPT-4 in brain tumors, ChatGPT-4o in hemorrhage detection) Supports automated cancer staging through TNM parameter extraction Aids in differential diagnosis and streamlines clinical workflow	Reduced performance for complex pathologies (e.g., CNS tumors, subarachnoid hemorrhages) Susceptible to errors such as misclassification or ambiguous/noncommittal outputs Requires well-crafted prompts for accurate results Risk of hallucinations and referencing errors Necessitates human review and oversight
**Report Generation**	Drafting key components of radiology reports: indication, findings, and impression Imaging recommendations Structured reporting	Generates diagnostic impressions based on imaging finding Enables structured, reproducible reporting Supports clinical decision-making by suggesting treatments and follow-up (e.g., orthopedics, oncology). Generating diagnostic impressions based on imaging finding Converting free-text reports into structured templates Multilingual and multimodal report standardization	May fall short of expert radiologist precision, especially in nuanced or rare cases Requires large, well-annotated training datasets for subspecialty fine-tuning Risk of overly generalized outputs, lacking clinical urgency or specificity Challenges in capturing language and context variability across clinical settings
**Patient Communication**	Patient-friendly summary generation	Enables tailored, easily comprehensible explanations of findings Enhances patient–provider communication Enhances patient understanding, engagement, and satisfaction	Risk of factual error propagation in AI-generated summaries Oversimplification may cause confusion or undue alarm Potential for patient distress or misguided self-directed action May undermine trust in medical professionals if inconsistently applied

**Table 3:** Clinical and research applications of LLMs in radiology, grouped by common functional domains to illustrate parallel use across practice and investigation.

Scenario	Clinical Applications	Research Applications
**Information Retrieval and Decision Support**	Clinical decision-making support (e.g., appropriateness criteria), imaging protocol selection	Literature review, guideline analysis, and identification of relevant studies or datasets
**Data Extraction and Structuring**	Extraction of clinical history from notes, structured report generation from free text	Automated extraction of variables from radiology reports and clinical notes for data analysis
**Image and Report Interpretation**	Multimodal report generation (e.g., combining image and text), impression summarization	Evaluation of multimodal LLMs for diagnostic accuracy and radiologic pattern recognition
**Communication and Language Simplification**	Patient-facing explanations of radiology reports, response generation for FAQs, multilingual support	Natural language generation for lay summaries, survey creation, and dissemination of results
**Analytical Processing and Computation**	Risk stratification tools, real-time response systems, model-assisted triage	Preliminary data cleaning, visualization, and statistical modeling using LLM-integrated environments
**Ethics, Bias and Sustainability**	Privacy-preserving model deployment, mitigation of clinical bias, responsible AI use in care settings	Study of bias propagation, environmental impact of training/deployment, evaluation of open-source model utility

## Data Availability

Not applicable.

## Abbreviations

The following abbreviations are used in this manuscript:

AI	Artificial intelligence
BERT	Bidirectional encoder representations from transformers
CAD-RADS	Coronary artery disease reporting and data system
CCTA	Coronary CT angiography
DWI	Diffusion-weighted imaging
GIA-CB	Gastrointestinal imaging-aware chatbot
GPT	Generative pretrained transformer
LLMs	Large language models
NLP	Natural language processing
RAG	Retrieval-augmented generation
USPSTF	United States Preventive Services Task Force
ViT	Vision transformer
ZSL	Zero-shot learning
